# Bis{4-[(2-hy­droxy-5-meth­oxy-3-nitro­benzyl­idene)amino]­phen­yl} ether

**DOI:** 10.1107/S2056989019016852

**Published:** 2020-01-01

**Authors:** Md. Azharul Arafath, Huey Chong Kwong, Farook Adam, Md. Mohiuddin, Md. Sohug Sarker, Mohammad Salim, Md. Mahbubul Alam

**Affiliations:** a Department of Chemistry, Shahjalal University of Science and Technology, Sylhet 3114, Bangladesh; b Department of Chemistry, Faculty of Science, Universiti Putra Malaysia, 43400 UPM Serdang, Selangor, Malaysia; c School of Chemical Sciences, Universiti Sains Malaysia, Penang 11800 USM, Malaysia

**Keywords:** crystal structure, oxybis Schiff base, inter­molecular inter­action

## Abstract

The mol­ecule of the title compound, C_28_H_22_N_4_O_9_, exhibits crystallographically imposed twofold rotational symmetry, with a dihedral angle of 66.0 (2)° between the planes of the two central benzene rings bounded to the central oxygen atom. The dihedral angle between the planes of the central benzene ring and the terminal phenol ring is 4.9 (2)°. Each half of the mol­ecule exhibits an imine *E* configuration. An intra­molecular O—H⋯N hydrogen bond is present. In the crystal, the mol­ecules are linked into layers parallel to the *ab* plane *via* C—H⋯O hydrogen bonds. The crystal studied was refined as a two-component pseudomerohedral twin.

## Abstract 2

The mol­ecule of the title oxybis compound lies on a twofold rotational axis. The conformation of the title compound is discussed and compared to those of related structures. In the crystal, mol­ecules of the title compound are assembled into layers parallel to the *ab* plane through C—H⋯O hydrogen bonds.

## Chemical context   

Bisthio­semicarbazones are formed by connecting separated thio­semicarbazone moieties through a pair of oxybisphenyl rings. These tetra­dentate ligands trap metals inside to form square-planar complexes (Alsop *et al.*, 2005 ▸; Blower *et al.*, 2003 ▸; Jasinski *et al.*, 2003 ▸). The length of the C—C bond in the backbone affects the stability of the complexes. A higher number of C—C bonds obtained *via* alkyl­ation or aryl­ation allows metal ions to better fit inside the ligand cavity (Blower *et al.*, 2003 ▸). These tetra­dentate ligands and transition-metal complexes exhibit promising anti­cancer and anti­bacterial activities (Lobana *et al.*, 2009 ▸). In view of this and our research inter­est in the synthesis of oxybis Schiff base compounds, we herein report the crystal structure, supra­molecular features and conformational comparison of the title compound.
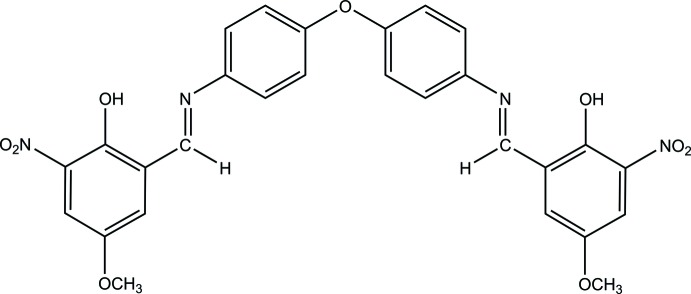


## Structural commentary   

**Figure 1:**
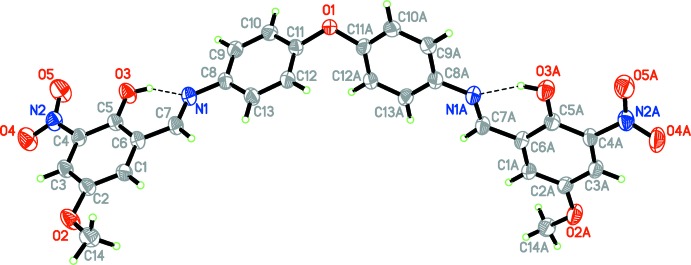
The mol­ecular structure of the title compound with displacement ellipsoids drawn at the 50% probability level. Intra­molecular hydrogen bonds are shown as dashed lines. Atoms with the label suffix A are generated by the symmetry operation −*x*, *y*, 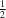 − *z*.

In the title compound (Fig. 1 ▸), the asymmetric unit comprises one half of the oxybisbenzenyl mol­ecule where the oxygen atom (O1) lies on a twofold rotation axis. The complete mol­ecule is generated through the symmetry operation −*x*, *y*, 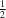 − *z*. The planes of the benzene rings bonded to the central oxygen atom form a dihedral angle of 66.0 (2)°. The dihedral angle between the benzene and 4-meth­oxy-2-nitro­phenol rings in the same half of the mol­ecules is 4.9 (2)°, indicating an almost coplanar arrangement of the benzene and phenol rings. The *sp* ^2^-hybridized character of atoms N1 and C7 is confirmed by the N1—C7 [1.287 (6) Å] bond length and C7—N1—C8 [121.9 (4)°] and N1—C7—C6 [121.7 (4)°] bond angles (Arafath *et al.*, 2018 ▸). Each half of the mol­ecule exhibits an imine *E* configuration with a C6—C7—N1—C8 torsion angle of 177.7 (4)°. In the mol­ecule, atom N1 of the imine moiety acts as a hydrogen-bond acceptor for the adjacent phenol group, forming an intra­molecular O—H⋯N hydrogen bond with an *S*(6) ring motif (Fig. 1 ▸, Table 1 ▸).

## Supra­molecular features   

**Figure 2:**
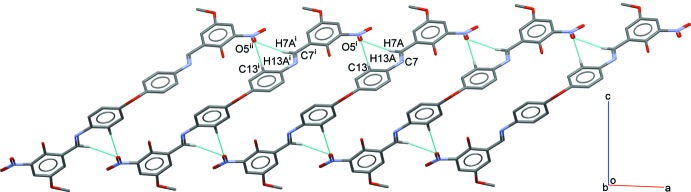
Partial packing diagram for the title compound, showing inter­molecular hydrogen bonds (cyan dotted lines). Hydrogen atoms not involved in hydrogen bonding are omitted for clarity. Symmetry codes: (i) −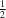 + *x*, 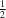 + *y*, *z*; (ii) −1 + *x*, 1 + *y*, *z*.

In the crystal, atom O5 acts as a bifurcated-hydrogen-bond acceptor, linking mol­ecules into layers parallel to the *ab* plane (Fig. 2 ▸) through C7—H7*A*⋯O5 and C13—H13*A*⋯O5 hydrogen bonds (Table 1 ▸). No C—H⋯π or π–π inter­actions are observed.

## Database survey   

**Figure 3:**
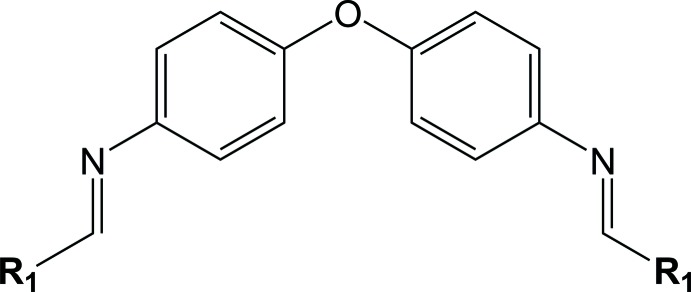
Structural fragment for the CSD search.

In a search of the Cambridge Structure Database (CSD, version 5.40, last update August 2019; Groom *et al.*, 2016 ▸), twelve structures containing the (1*E*,1′*E*)-*N*,*N*′-[oxybis(4,1-phenyl­ene)]bis­(1-phenyl­methanimine) moiety with different substituents were found. The reference moiety is illustrated in Fig. 3 ▸. Details regarding different substituents (**R_1_**) together with the dihedral and torsion angles for oxybisbenzenyl moiety in these structures are tabulated in Table 2 ▸. In analogy with the title mol­ecule, the planes of the central benzene ring bonded to the central oxygen atom are always V-shaped with dihedral angle 1 in the range of 54.6–84.8°. The dihedral angle between the planes of central and terminal benzene rings exists in two conformations, *viz*. non-coplanar [dihedral 2 = 18.0–73.5°] and nearly coplanar [dihedral 2 = 4.8–9.9°]. In all of these structures, the imine C=N double bond adopts an *E* configuration with torsion angles corresponding to C6—C7—N1—C8 in the range 172.9–180.0°.

## Synthesis and crystallization   

**Figure 4:**
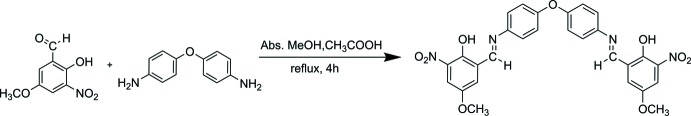
Reaction scheme for the synthesis of the title compound.

To a sample of 2-hy­droxy-5-meth­oxy-3-nitro­benzaldehyde (0.98 g, 5.00 mmol) dissolved in 25.0 mL of methanol, 0.20 mL of glacial acetic acid were added, and the mixture was refluxed for 30 min. A solution of 4,4′-oxydianiline (0.50 g, 2.50 mmol) in 20.0 mL of methanol was added dropwise under stirring to the aldehyde solution. The resulting deep-red solution was refluxed for 4 h with stirring. The reaction scheme is shown in Fig. 4 ▸. The deep-red precipitate that formed was filtered off and washed with 5.0 mL of methanol and 5.0 mL of *n*-hexane. The recovered product was dissolved in chloro­form for recrystallization. Purple single crystals suitable for X-ray diffraction were obtained by slow evaporation of the solvent, m.p. 547–548 K, yield 96%. Analysis calculated for C_28_H_22_N_4_O_9_ (f.w. 558.50 g mol^−1^) C, 60.16; H, 3.93; N, 10; found: C, 59.04; H, 3.85; N, 9.90%. ^1^H NMR (500 MHz, DMSO-*d*_6_, Me_4_Si ppm): δ 10.23 (*s*, OH), δ 9.12 (*s*, HC=N), δ 7.69–7.21 (multiplet, aromatic), δ 3.83 (*s*, Ph—OCH_3_). ^13^C NMR (DMSO-*d*_6_, Me_4_Si ppm): δ 161.69 (C=N), δ 156.21–114.96 (C-aromatic), δ 56.25 (OCH_3_). IR (KBr pellets υ_max_/cm^−1^): 3441 υ(OH), 3109 υ(C—H, *sp* ^2^), 2956 υ(CH_3_), 1598 υ(C=N), 1529 υ(C=C, aromatic), 1497 υ(NO_2_, asym.), 1326 υ(NO_2_, sym.), 1257 υ(C—O, phenolic), 1194 υ(C—O, Ph—OCH_3_), 1056 υ(C—N), 979 υ(CH, bend. aromatic).

## Refinement   

Crystal data, data collection and structure refinement details are summarized in Table 3 ▸. The phenolic hydrogen atom was located in a difference-Fourier map and refined freely. All other H atoms attached to C were positioned geometrically and refined using a riding model with C—H= 0.95–0.98 Å and *U*_iso_(H) = 1.2*U*_eq_(C) or 1.5*U*_eq_(C) for methyl H atoms. A rotating model was used for the methyl group. The crystal investigated was refined as a two-component pseudomerohedral twin resulting from a 180° rotation about the [001] reciprocal lattice direction, with a twin ratio of 0.977 (3):0.023 (3).

## Supplementary Material

Crystal structure: contains datablock(s) I. DOI: 10.1107/S2056989019016852/rz5267sup1.cif

Structure factors: contains datablock(s) I. DOI: 10.1107/S2056989019016852/rz5267Isup2.hkl

Supporting information file. DOI: 10.1107/S2056989019016852/rz5267Isup3.cml

CCDC reference: 1445336

Additional supporting information:  crystallographic information; 3D view; checkCIF report

## Figures and Tables

**Table 1:** Hydrogen-bond geometry (Å, °)

*D*—H⋯*A*	*D*—H	H⋯*A*	*D*⋯*A*	*D*—H⋯*A*
O3—H1*O*3⋯N1	0.85 (9)	1.81 (10)	2.591 (6)	153 (7)
C7—H7*A*⋯O5^i^	0.95	2.54	3.470 (7)	167
C13—H13*A*⋯O5^i^	0.95	2.48	3.404 (7)	165

Symmetry code: (i) 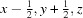.

**Table 2:** Selected dihedral and torsion angles (°) Dihedral 1 is the dihedral angle between the planes of the central benzene rings. Dihedral 2 is the dihedral angle between the planes of the central and terminal benzene rings.

Compound	***R*_1_**	Dihedral 1	Dihedral 2	C6—C7—N1—C8
(I)	4-meth­oxy-2-nitro­phenol	66.0 (2)	4.9 (2), 4.9 (2)	−177.7 (4), −177.7 (4)
DICKUW (Chu & Huang, 2007 ▸)	2,4-di-*tert*-butyl­phenol	73.8	4.8, 35.5	178.2, 177.2
DICLAD (Chu & Huang, 2007 ▸)	2-(*tert*-but­yl)-4-methyl­phenol	73.8	47.9, 46.3	175.2, −179.9
GIFCEG (Arafath *et al.*, 2018 ▸)	2-methyl­phenol	59.5	36.0, 31.5	178.3, 179.0
HUDJEW (Lee & Lee, 2009 ▸)	4-nitro­phen­yl	75.7	53.0, 18.0	−174.0, 179.2
NATWEM (Khalaji *et al.*, 2012 ▸)	2,3,4-tri­meth­oxy­phen­yl	84.8	57.6, 73.1	−179.2, −175.7
PEHGOA (Kadu *et al.*, 2013 ▸)	phen­yl	59.8	8.8, 6.0	−179.9, 179.8
PEHHAN (Kadu *et al.*, 2013 ▸)	4-meth­oxy­phen­yl	60.1	5.3, 5.3	−179.3, −179.3
RIZFEM (Xu *et al.*, 2008 ▸)	2-meth­oxy­phenol	69.2	24.3, 24.3	−180.0, −180.0
TOWSOP (Kaabi *et al.*, 2015 ▸)	3-(di­ethyl­amino)­phenol	65.7	41.4, 30.6	−173.1, −176.5
UNUFEP (Shahverdizadeh & Tiekink, 2011 ▸)	phenol	54.6	51.6, 51.6	173.5, 173.4
WEFLUQ (Krishna *et al.*, 2012 ▸)	naphthalen-2-ol	75.1/70.1	7.7, 9.9/6.1, 19.4	176.5, 177.6/-179.3, −172.9
WIGPOT (Haffar *et al.*, 2013 ▸)	naphthalen-2-ol	74.6/69.9	7.7. 9.9/19.6, 5.8	177.2, 176.3/ −172.9, −178.6

Note: there is more than one data set for compounds WEFLUQ and WIGPOT because there is more than one independent mol­ecule in their asymmetric units.

**Table 3:** Experimental details

Crystal data	
Chemical formula	C_28_H_22_N_4_O_9_
*M*_r_	558.49
Crystal system, space group	Monoclinic, *C*2/*c*
Temperature (K)	100
*a*, *b*, *c* (Å)	15.954 (4), 5.4599 (12), 28.397 (6)
β (°)	92.299 (5)
*V* (Å^3^)	2471.7 (10)
*Z*	4
Radiation type	Mo *K*α
μ (mm^−1^)	0.11
Crystal size (mm)	0.38 × 0.24 × 0.14

Data collection	
Diffractometer	Bruker APEX DUO CCD area detector
Absorption correction	Multi-scan (*SADABS*; Bruker, 2012 ▸)
*T*_min_, *T*_max_	0.879, 0.956
No. of measured, independent and observed [*I* > 2σ(*I*)] reflections	35811, 2830, 2591
*R*_int_	0.038
(sin θ/λ)_max_ (Å^−1^)	0.650

Refinement	
*R*[*F* ^2^ > 2σ(*F* ^2^)], *wR*(*F* ^2^), *S*	0.100, 0.353, 1.15
No. of reflections	2830
No. of parameters	192
H-atom treatment	H atoms treated by a mixture of independent and constrained refinement
Δρ_max_, Δρ_min_ (e Å^−3^)	0.31, −0.31

Computer programs: *APEX2* and *SAINT* (Bruker, 2012 ▸), *SHELXS97* (Sheldrick, 2008 ▸), *SHELXL2013* (Sheldrick, 2015 ▸), *Mercury* (Macrae *et al.*, 2006 ▸) and *PLATON* (Spek, 2009 ▸).

## supplementary crystallographic information

### Crystal data

**Table:**

C_28_H_22_N_4_O_9_	*F*(000) = 1160
*M**_r_* = 558.49	*D*_x_ = 1.501 Mg m^−^^3^
Monoclinic, *C*2/*c*	Mo *K*α radiation, λ = 0.71073 Å
*a* = 15.954 (4) Å	Cell parameters from 9905 reflections
*b* = 5.4599 (12) Å	θ = 3–31°
*c* = 28.397 (6) Å	µ = 0.11 mm^−^^1^
β = 92.299 (5)°	*T* = 100 K
*V* = 2471.7 (10) Å^3^	Block, purple
*Z* = 4	0.38 × 0.24 × 0.14 mm

### Data collection

**Table:**

Bruker APEX DUO CCD area detector diffractometer	2830 independent reflections
Radiation source: fine-focus sealed tube	2591 reflections with *I* > 2σ(*I*)
Graphite monochromator	*R*_int_ = 0.038
φ and ω scans	θ_max_ = 27.5°, θ_min_ = 0.7°
Absorption correction: multi-scan (SADABS; Bruker, 2012)	*h* = −20→20
*T*_min_ = 0.879, *T*_max_ = 0.956	*k* = −7→7
35811 measured reflections	*l* = −36→36

### Refinement

**Table:**

Refinement on *F*^2^	0 restraints
Least-squares matrix: full	Hydrogen site location: mixed
*R*[*F*^2^ > 2σ(*F*^2^)] = 0.100	H atoms treated by a mixture of independent and constrained refinement
*wR*(*F*^2^) = 0.353	*w* = 1/[σ^2^(*F*_o_^2^) + (0.1539*P*)^2^ + 17.7934*P*] where *P* = (*F*_o_^2^ + 2*F*_c_^2^)/3
*S* = 1.15	(Δ/σ)_max_ < 0.001
2830 reflections	Δρ_max_ = 0.31 e Å^−^^3^
192 parameters	Δρ_min_ = −0.31 e Å^−^^3^

### Special details

**Table:**

Experimental. The following wavelength and cell were deduced by SADABS from the direction cosines etc. They are given here for emergency use only: CELL 0.71095 5.463 8.443 28.418 92.106 89.981 108.897
Geometry. All esds (except the esd in the dihedral angle between two l.s. planes) are estimated using the full covariance matrix. The cell esds are taken into account individually in the estimation of esds in distances, angles and torsion angles; correlations between esds in cell parameters are only used when they are defined by crystal symmetry. An approximate (isotropic) treatment of cell esds is used for estimating esds involving l.s. planes.
Refinement. Refined as a 2-component twin.

### Fractional atomic coordinates and isotropic or equivalent isotropic displacement parameters (Å^2^)

**Table:**

	*x*	*y*	*z*	*U*_iso_*/*U*_eq_	
O1	0.000000	−0.2692 (9)	0.250000	0.0444 (13)	
O2	0.4044 (2)	0.9524 (8)	0.47761 (16)	0.0525 (11)	
O3	0.4346 (3)	0.1480 (8)	0.36154 (14)	0.0475 (10)	
O4	0.6359 (2)	0.4545 (9)	0.43220 (19)	0.0664 (14)	
O5	0.5853 (3)	0.1292 (9)	0.4007 (2)	0.0710 (15)	
N1	0.2747 (2)	0.1732 (8)	0.34224 (14)	0.0371 (9)	
N2	0.5771 (3)	0.3336 (9)	0.41607 (16)	0.0417 (10)	
C1	0.3380 (3)	0.6629 (9)	0.42249 (18)	0.0364 (10)	
H1A	0.284746	0.739136	0.424432	0.044*	
C2	0.4061 (3)	0.7544 (9)	0.44839 (17)	0.0355 (10)	
C3	0.4833 (3)	0.6438 (9)	0.44506 (17)	0.0365 (10)	
H3A	0.530272	0.707394	0.462653	0.044*	
C4	0.4934 (3)	0.4407 (9)	0.41635 (16)	0.0337 (10)	
C5	0.4255 (3)	0.3424 (9)	0.38929 (16)	0.0332 (10)	
C6	0.3471 (3)	0.4586 (9)	0.39343 (16)	0.0337 (10)	
C7	0.2723 (3)	0.3645 (9)	0.36861 (17)	0.0366 (10)	
H7A	0.220289	0.446315	0.371966	0.044*	
C8	0.2016 (3)	0.0754 (9)	0.31932 (16)	0.0335 (10)	
C9	0.2115 (3)	−0.1371 (9)	0.29336 (17)	0.0366 (10)	
H9A	0.265646	−0.208522	0.291709	0.044*	
C10	0.1439 (3)	−0.2462 (9)	0.26992 (16)	0.0369 (10)	
H10A	0.151248	−0.392646	0.252498	0.044*	
C11	0.0657 (3)	−0.1405 (9)	0.27200 (16)	0.0349 (10)	
C12	0.0535 (3)	0.0722 (9)	0.29753 (18)	0.0395 (11)	
H12A	−0.000735	0.142841	0.298889	0.047*	
C13	0.1217 (3)	0.1799 (9)	0.32098 (17)	0.0386 (11)	
H13A	0.114209	0.326303	0.338386	0.046*	
C14	0.3252 (3)	1.0498 (11)	0.4876 (2)	0.0477 (13)	
H14A	0.332369	1.185096	0.510095	0.072*	
H14B	0.290647	0.921682	0.501263	0.072*	
H14C	0.297545	1.110264	0.458451	0.072*	
H1O3	0.386 (6)	0.117 (15)	0.350 (3)	0.08 (3)*	

### Atomic displacement parameters (Å^2^)

**Table:**

	*U*^11^	*U*^22^	*U*^33^	*U*^12^	*U*^13^	*U*^23^
O1	0.047 (3)	0.029 (2)	0.055 (3)	0.000	−0.023 (2)	0.000
O2	0.0300 (17)	0.054 (2)	0.073 (3)	0.0037 (16)	−0.0050 (16)	−0.031 (2)
O3	0.0378 (19)	0.054 (2)	0.050 (2)	0.0037 (16)	−0.0020 (15)	−0.0231 (18)
O4	0.0290 (18)	0.074 (3)	0.095 (3)	0.0049 (19)	−0.013 (2)	−0.024 (3)
O5	0.044 (2)	0.069 (3)	0.099 (4)	0.018 (2)	−0.008 (2)	−0.039 (3)
N1	0.0291 (18)	0.043 (2)	0.039 (2)	−0.0034 (16)	−0.0033 (15)	−0.0025 (17)
N2	0.0296 (19)	0.052 (2)	0.044 (2)	0.0046 (18)	0.0000 (16)	−0.0073 (19)
C1	0.027 (2)	0.035 (2)	0.047 (2)	0.0005 (17)	−0.0033 (18)	−0.003 (2)
C2	0.030 (2)	0.037 (2)	0.039 (2)	−0.0011 (18)	0.0016 (17)	−0.0075 (19)
C3	0.027 (2)	0.041 (2)	0.042 (2)	−0.0031 (18)	−0.0018 (17)	−0.006 (2)
C4	0.0241 (19)	0.041 (2)	0.036 (2)	0.0020 (17)	−0.0006 (16)	−0.0026 (18)
C5	0.030 (2)	0.037 (2)	0.033 (2)	−0.0002 (18)	0.0015 (16)	−0.0051 (18)
C6	0.028 (2)	0.040 (2)	0.033 (2)	−0.0040 (18)	−0.0020 (16)	−0.0031 (18)
C7	0.027 (2)	0.043 (3)	0.040 (2)	−0.0021 (18)	−0.0027 (17)	−0.003 (2)
C8	0.031 (2)	0.037 (2)	0.032 (2)	−0.0019 (18)	−0.0028 (16)	0.0004 (18)
C9	0.034 (2)	0.036 (2)	0.039 (2)	0.0035 (18)	−0.0025 (18)	−0.0007 (19)
C10	0.042 (2)	0.033 (2)	0.035 (2)	0.0023 (19)	−0.0030 (18)	−0.0017 (18)
C11	0.039 (2)	0.034 (2)	0.032 (2)	−0.0052 (18)	−0.0086 (17)	0.0028 (18)
C12	0.034 (2)	0.037 (2)	0.046 (3)	0.0053 (19)	−0.0107 (19)	−0.004 (2)
C13	0.037 (2)	0.038 (2)	0.041 (2)	0.0022 (19)	−0.0072 (18)	−0.011 (2)
C14	0.037 (2)	0.046 (3)	0.060 (3)	0.007 (2)	0.003 (2)	−0.017 (3)

### Geometric parameters (Å, º)

**Table:**

O1—C11^i^	1.389 (5)	C4—C5	1.409 (6)
O1—C11	1.389 (5)	C5—C6	1.412 (6)
O2—C2	1.364 (6)	C6—C7	1.456 (6)
O2—C14	1.410 (6)	C7—H7A	0.9500
O3—C5	1.333 (6)	C8—C9	1.387 (7)
O3—H1O3	0.85 (9)	C8—C13	1.398 (6)
O4—N2	1.221 (6)	C9—C10	1.380 (7)
O5—N2	1.207 (6)	C9—H9A	0.9500
N1—C7	1.287 (6)	C10—C11	1.379 (7)
N1—C8	1.418 (6)	C10—H10A	0.9500
N2—C4	1.458 (6)	C11—C12	1.387 (7)
C1—C2	1.381 (6)	C12—C13	1.384 (6)
C1—C6	1.398 (7)	C12—H12A	0.9500
C1—H1A	0.9500	C13—H13A	0.9500
C2—C3	1.377 (6)	C14—H14A	0.9800
C3—C4	1.390 (7)	C14—H14B	0.9800
C3—H3A	0.9500	C14—H14C	0.9800
			
C11^i^—O1—C11	119.2 (5)	N1—C7—H7A	119.2
C2—O2—C14	117.5 (4)	C6—C7—H7A	119.2
C5—O3—H1O3	106 (6)	C9—C8—C13	118.9 (4)
C7—N1—C8	121.9 (4)	C9—C8—N1	116.7 (4)
O5—N2—O4	122.8 (5)	C13—C8—N1	124.4 (4)
O5—N2—C4	119.0 (4)	C10—C9—C8	120.9 (4)
O4—N2—C4	118.1 (4)	C10—C9—H9A	119.6
C2—C1—C6	120.3 (4)	C8—C9—H9A	119.6
C2—C1—H1A	119.8	C11—C10—C9	119.4 (4)
C6—C1—H1A	119.8	C11—C10—H10A	120.3
O2—C2—C3	115.4 (4)	C9—C10—H10A	120.3
O2—C2—C1	125.2 (4)	C10—C11—C12	121.2 (4)
C3—C2—C1	119.4 (4)	C10—C11—O1	115.9 (4)
C2—C3—C4	121.0 (4)	C12—C11—O1	122.7 (4)
C2—C3—H3A	119.5	C13—C12—C11	118.9 (4)
C4—C3—H3A	119.5	C13—C12—H12A	120.5
C3—C4—C5	121.3 (4)	C11—C12—H12A	120.5
C3—C4—N2	116.8 (4)	C12—C13—C8	120.7 (4)
C5—C4—N2	121.9 (4)	C12—C13—H13A	119.7
O3—C5—C4	121.8 (4)	C8—C13—H13A	119.7
O3—C5—C6	121.6 (4)	O2—C14—H14A	109.5
C4—C5—C6	116.6 (4)	O2—C14—H14B	109.5
C1—C6—C5	121.4 (4)	H14A—C14—H14B	109.5
C1—C6—C7	117.7 (4)	O2—C14—H14C	109.5
C5—C6—C7	120.9 (4)	H14A—C14—H14C	109.5
N1—C7—C6	121.7 (4)	H14B—C14—H14C	109.5
			
C14—O2—C2—C3	−170.9 (5)	O3—C5—C6—C7	2.1 (7)
C14—O2—C2—C1	9.8 (8)	C4—C5—C6—C7	−177.2 (4)
C6—C1—C2—O2	179.7 (5)	C8—N1—C7—C6	177.7 (4)
C6—C1—C2—C3	0.5 (8)	C1—C6—C7—N1	−178.0 (5)
O2—C2—C3—C4	−179.8 (5)	C5—C6—C7—N1	0.1 (7)
C1—C2—C3—C4	−0.5 (8)	C7—N1—C8—C9	−177.5 (4)
C2—C3—C4—C5	0.7 (7)	C7—N1—C8—C13	3.2 (8)
C2—C3—C4—N2	−178.6 (5)	C13—C8—C9—C10	−0.7 (7)
O5—N2—C4—C3	163.3 (5)	N1—C8—C9—C10	179.9 (4)
O4—N2—C4—C3	−15.5 (7)	C8—C9—C10—C11	0.7 (7)
O5—N2—C4—C5	−16.1 (8)	C9—C10—C11—C12	−0.5 (7)
O4—N2—C4—C5	165.1 (5)	C9—C10—C11—O1	−176.1 (4)
C3—C4—C5—O3	179.8 (5)	C11^i^—O1—C11—C10	−145.5 (5)
N2—C4—C5—O3	−0.9 (7)	C11^i^—O1—C11—C12	39.0 (4)
C3—C4—C5—C6	−0.8 (7)	C10—C11—C12—C13	0.4 (8)
N2—C4—C5—C6	178.5 (4)	O1—C11—C12—C13	175.7 (4)
C2—C1—C6—C5	−0.7 (7)	C11—C12—C13—C8	−0.5 (8)
C2—C1—C6—C7	177.4 (5)	C9—C8—C13—C12	0.6 (8)
O3—C5—C6—C1	−179.8 (5)	N1—C8—C13—C12	180.0 (5)
C4—C5—C6—C1	0.8 (7)		

Symmetry code: (i) −*x*, *y*, −*z*+1/2.

### Hydrogen-bond geometry (Å, º)

**Table:**

*D*—H···*A*	*D*—H	H···*A*	*D*···*A*	*D*—H···*A*
O3—H1*O*3···N1	0.85 (9)	1.81 (10)	2.591 (6)	153 (7)
C7—H7*A*···O5^ii^	0.95	2.54	3.470 (7)	167
C13—H13*A*···O5^ii^	0.95	2.48	3.404 (7)	165

Symmetry code: (ii) *x*−1/2, *y*+1/2, *z*.
